# Non-viral vector based gene transfection with human induced pluripotent stem cells derived cardiomyocytes

**DOI:** 10.1038/s41598-019-50980-w

**Published:** 2019-10-07

**Authors:** Shihua Tan, Zhonghao Tao, Szejie Loo, Liping Su, Xin Chen, Lei Ye

**Affiliations:** 1 National Heart Research Institute Singapore, National Heart Centre Singapore, Singapore, Singapore; 2 Department of Thoracic and Cardiovascular Surgery, Nanjing First Hospital, Nanjing Medical University, Nanjing, Jiangsu China

**Keywords:** Genetic vectors, Angiogenesis

## Abstract

Non-viral transfection of mammalian cardiomyocytes (CMs) is challenging. The current study aims to characterize and determine the non-viral vector based gene transfection efficiency with human induced pluripotent stem cells (hiPSCs) derived cardiomyocytes (hiPSC-CMs). hiPSC-CMs differentiated from PCBC hiPSCs were used as a cell model to be transfected with plasmids carrying green fluorescence protein (pGFP) using polyethylenimine (PEI), including Transporter 5 Transfection Reagent (TR5) and PEI25, and liposome, including lipofectamine-2000 (Lipo2K), lipofectamine-3000 (Lipo3K), and Lipofectamine STEM (LipoSTEM). The gene transfection efficiency and cell viability were quantified by flow cytometry. We found that the highest gene transfection efficiency in hiPSC-CMs on day 14 of contraction can be achieved by LipoSTEM which was about 32.5 ± 6.7%. However, it also cuased poor cell viability (60.1 ± 4.5%). Furthermore, a prolonged culture of (transfection on day 23 of contraction) hiPSC-CMs not only improved gene transfection (54.5 ± 8.9%), but also enhanced cell viability (74 ± 4.9%) by LipoSTEM. Based on this optimized gene transfection condition, the highest gene transfection efficiency was 55.6 ± 7.8% or 34.1 ± 4%, respectively, for P1C1 or DP3 hiPSC line that was derived from healthy donor (P1C1) or patient with diabetes (DP3). The cell viability was 80.8 ± 5.2% or 92.9 ± 2.24%, respectively, for P1C1 or DP3. LipoSTEM is a better non-viral vector for gene transfection of hiPSC-CMs. The highest pGFP gene transfection efficiency can reach >50% for normal hiPSC-CMs or >30% for diabetic hiPSC-CMs.

## Introduction

Cardiovascular disease is a major cause of mortality throughout the world. In addition to pharmacologic drugs and device therapies, gene-based angiogenic therapies for treatment of heart failure are tested in hopes of being translated to the clinical setting^1^. Animal studies showed that myocardial gene transfer of vascular endothelial growth factor (VEGF) or fibroblast growth factor (FGF) can improve cardiac angiogenesis^2,3^ or connexin-43 can limit atrial fibrillation and ventricular tachycardia^4^. Mammalian cardiomyocytes (CMs) are terminally differentiated somatic cells. So far, only viruses, including adenovirus, adeno-associated virus, and lentivirus, have successfully transduced CMs for high gene transduction efficiencies^3,5–9^. Non-viral vector based gene transfection with CMs is very challenging.

Our previous study showed that polyethyleminine (PEI) or liposome based non-viral vector can efficiently transfect human skeletal myoblasts which transiently expressed VEGF protein for 14 days^10–12^. In the current study, we aim to characterize non-viral vector based gene transfer with human induced pluripotent stem cells (hiPSCs) derived CMs (hiPSC-hiPSC-CMs). hiPSCs, which are reprogrammed from adult human somatic cells by defined transcription factors, have emerged as a better alternative for deriving autologous hiPSC-CMs. Using established differentiation protocols^13–15^, a large amount of CMs can be generated from hiPSCs and are being tested as cell transfer therapy for cardiac repair in animal models^16^. We found that Lipofectamine-STEM (LipoSTEM) based non-viral vector transfection achieved a higher gene transfection efficiency than PEI using plasmids carrying green fluorescence protein (pGFP).

## Methods

### Human iPSC generation

Three hiPSC lines used in this study were reprogrammed from dermal fibroblasts using non-integrating Sendai virus and the reprogramming factors OCT4, SOX2, KLF4, and C-MYC, as described previously^13,17^. PCBC and P1C1 were reprogrammed from neonatal human dermal fibroblasts (Lonza, USA)^18^. DP3 was reprogrammed from dermal fibroblasts isolated from a patient with type 2 diabetes mellitus (T2DM)^17^.

### Culture and differentiation of human induced pluripotent stem cells

A well established hiPSC line, PCBC, was used as a cell model for gene transfection with pGFP. hiPSCs were cultured as a monolayer on growth factor reduced Matrigel coated surface in mTeSR/E8 (1:1) media (STEM CELL Tech., Canada). The differentiation protocol of hiPSCs into cardiomyocytes was described by Lian *et al*.^19^. Briefly, hiPSCs were dissociated into single cells with Versene (Thermo Fisher, USA) and cultured in mTeSR/E8 media for 4–5 days until confluence. On day 0, hiPSCs would be cultured in RPMI medium supplemented with 1x B27 without insulin (B27-) and 10 μM CHIR99021 for 24 hrs. On day 1, differentiation medium will be changed to RPMI/B27- for 48 hrs. On day 3 cells would be cultured in RPMI/B27- supplemented with 4 μM IWP2 for 48 hrs. On day 5, differentiation medium would be changed to RPMI/B27- for 48 hrs. On day 7 of differentiation and every 3 d thereafter, cell culture medium would be change to RPMI/B27 medium. Generally, differentiated hiPSCs would start contracting between day 8–10 of differentiation.

### Purification of hiPSC-CMs

**Figure 1:**
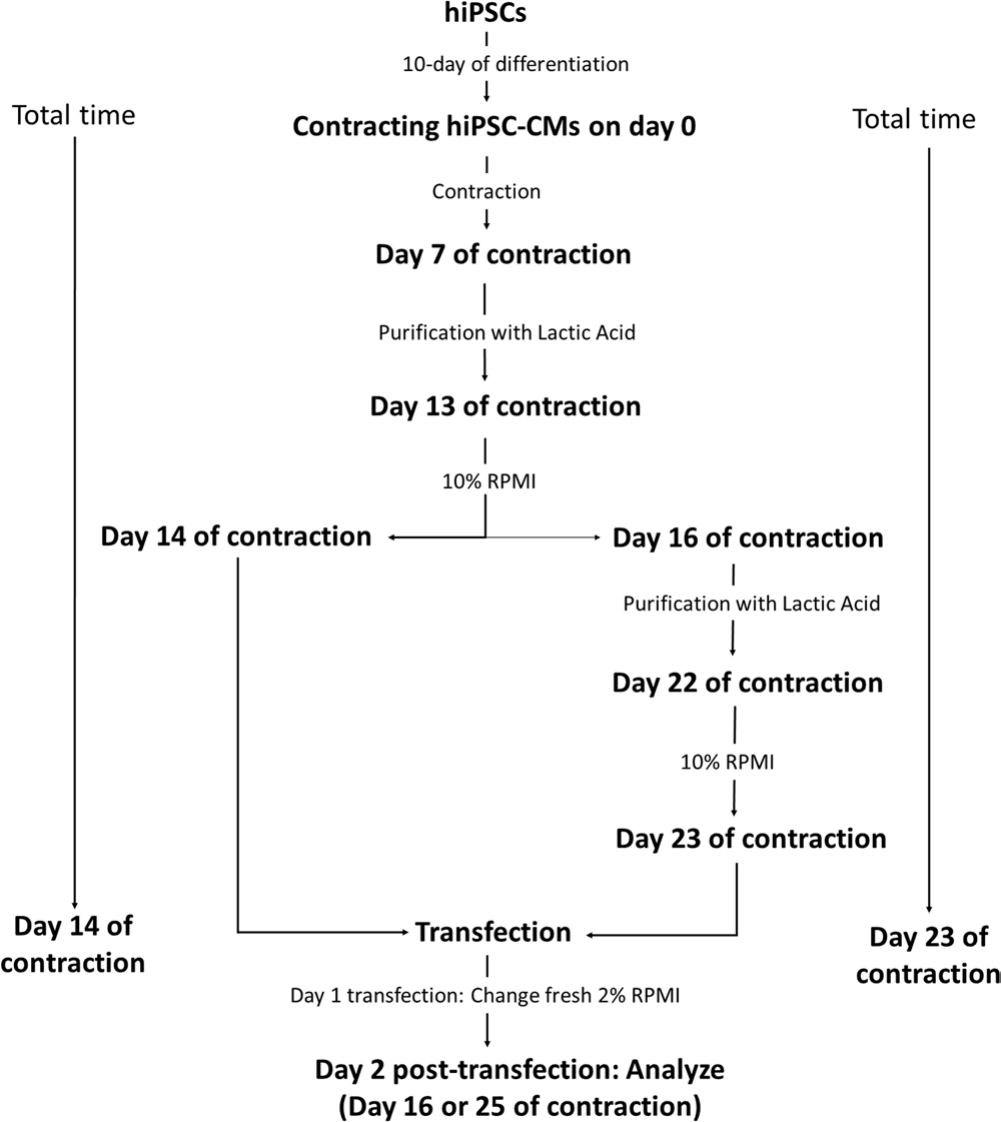
A schematic diagram of the hiPSC-CMs undergo purification and transfection.

The days of purification and transfection of hiPSC-CMs are listed in Fig. 1. To exclude non-CMs, we dissociated hiPSC-CMs into single cells on day 7 of contraction and cultured cells in RPMI medium without glucose (RPMI-), but supplemented with 1X non-essential amino acids (NEAA), 1X L-Glutamine GlutaMAX, 1X Antibiotic-Antimycotic, 55 nM β-mercaptoethanol (all from Thermo Fisher, USA), and 4 mM lactic acid (Sigma Aldrich, USA)^20^ for 6 days. On day 13 of contraction, purified cells would be 1. cultured in RPMI medium supplemented with 10% FBS (10%RPMI) for 24 hr and subsequently harvested for gene transfection with pGFP on day 14 of contraction or 2. continuously cultured in 10% RPMI for 3 days (till day 16 of contraction) and replaced with lactic acid medium for another 6 days of purification (till day 22 of contraction), followed by 10% RPMI for 24 hr. Cells would be harvested for gene transfection with pGFP on day 23 of contraction.

### Fluorescence immunostaining

Purified and non-purified hiPSC-CMs were harvested and fixed with 1% paraformaldehyde for 10 minutes at 37 °C. Cells were then permeabilized using 90% ice cold methanol for 30 minutes on ice. Ultra V Block (Thermo Fisher, USA) was added to the cells for blocking and incubated for 7 minutes. Primary antibody Anti-Cardiac Troponin T (564766, BD Biosciences, USA) was incubated with cells at 4 °C overnight while secondary antibody donkey anti-mouse Alexa Fluor ® 555 (1:400 dilution) (A31570, Thermo Fisher, USA) was incubated with cells for 30 minutes at room temperature. The stained hiPSC-CMs with adequate size and granularity were included in the statistical analysis for determining the purity of hiPSC-CMs.

### Preparation of polyethyleminine and polyplexes with pGFP

Plasmids carrying GFP described previously was used in this study^10^. Transporter™ 5 Transfection Reagent (TR5), which is made from linear PEI, was purchased from Polysciences Inc. (USA), while PEI of 25kD (PEI25) was purchased from Santa Cruz Biotech.,(USA). PEI25 was diluted in distilled water to make a 17 μM stock solution and passed through a 0.22 μm filter. 1 μL of 17 μM PEI25 contains 10 mM nitrogen residues. The mixture of TR5 to DNA was based on volume of TR5: quantity of plasmid DNA as per data sheet. The mixture of PEI25 to DNA was based on the equivalents of PEI nitrogen per DNA phosphate (N/P)^10,11^. pGFP and PEI25 or TR5 were diluted in 25 μl of 150 mM NaCl separately. Polyplexes were developed by mixing the respective 150 mM NaCl solutions containing PEI25/TR5 or pGFP. After complexation, the mixture was vortexed for 30 sec followed by sedation for 20 min. Then, the polyplex mixture was added to suspended hiPSC-CMs in RPMI and incubated at room temperature for 10 min. RPMI supplemented with 4% FBS (4% RPMI) was added to make a final concentration of 2%FBS in RPMI (2% RPMI) and mixture was seeded into Matrigel coated 12-well plastic plates for transfection with hiPSC-CMs over a 24 hr period at 37 °C in incubator.

### Preparation of liposome and lipoplexes with pGFP

Lipofectamine 2000 (Lipo2k), Lipofectamine 3000 (Lipo3k), and LipoSTEM were purchased from Thermo Fisher. The liposomes and plasmid DNA were each diluted in 25 μl of 150 mM NaCl, separately. The lipoplexes were developed based on the volume of liposomes: quantity of pGFP by mixing the respective solutions containing liposomes and plasmid DNA as per reagent instruction. After complexation, the mixture was vortexed for 5 sec followed by sedation for 10 min. Then, the lipoplex mixture was added to suspended hiPSC-CMs in RPMI and incubated at room temperature for 10 min. 4% RPMI was added to make a final concentration of 2% RPMI and mixture was seeded into Matrigel coated 12-well plastic plates for transfection over a 24 hr period at 37 °C in incubator.

### Cardiomyocyte gene transfection with pGFP

Trypsinized hiPSC-CMs on days 14 or 23 of contraction were seeded at a density of 2 × 10^5^ cells/well in 12-well plates.

#### Transfection of CMs using polyplexes

For TR5, the transfection ratio was based on volume of TR5 (μl) and quantity of pGFP (μg) and was tested from 1:1 to 3:1. For PEI25, the N/P ratio was tested from 3:1 to 9:1. The polyplexes were developed as described above and added into culture medium to transfect suspended hiPSC-CMs for 24 hr at 37 °C.

#### Transfection of CMs using lipoplexes

The transfection ratio was based on the volume of liposome (μl) and quantity of pGFP (μg). For Lipo2k, the transfection ratio was tested from 1:1 to 1:3. For Lipo3k, the transfection ratio was tested from 0.75:0.5 to 1.5:0.5. For LipoSTEM, the transfection ratio was tested at 0.9:0.3 and 1.2:0.4, then increased pGFP from 0.4 to 1.2 with fixed LipoSTEM at 1.2 μl. Lipoplexes were developed as described above and added into culture medium for transfection with suspended hiPSC-CMs for 24 hr at 37 °C in incubator. Olympus IX73 microscope and Cell Sens Standard software (both from Olympus) were used for imaging protein expression of GFP.

#### Transfection efficiency and cell viability

Transfection efficiency and cell viability of hiPSC-CMs were analyzed by flow cytometry using BD LSR II (BD Biosciences, USA) on day 2 post pGFP transfection. The transfection medium was collected and the cells were washed with phosphate-buffered saline (PBS) and harvested by 0.25% trypsin. The hiPSC-CMs expressing GFP with adequate size and granularity were included in the statistical analysis for assessing gene transfection efficiency and cell viability^10,11^. The total events for each flow analysis is 10,000. Acquired data was analyzed with FlowJo Version 7.6.2 (Treestar Software, Ashland, OR, USA).

### Cardiomyocyte gene transfection with angiopoietin-1 (Ang-1) plasmids (pAng-1)

We transfected hiPSC-CM on day 23 of contraction using LipoSTEM based on optimized transfection ratio. The size of pAng-1 is 7401 bp with an EF-1α promoter. To quantify Ang-1 concentration in supernanant secreted by Ang-1 transfected or non-transfected hiPSC-CMs, 2 × 10^5^ cells/well were grown **in** 12-well plate and the cell supernatant samples were collected on day 2 after transfection. Human Ang-1 Sandwich ELISA kit (Abbexa, USA) was used to quantify Ang-1 protein in supernantant according to supplier’s instructions.

### Statistical analysis

Statistical analysis was performed using SPSS (version 18.0). All data were presented as mean ± standard deviation (SD). Comparisons among groups were analyzed for significance via one-way analysis of variance (ANOVA) with the Tukey correction. P < 0.05 was considered as statistical significance.

## Results

### Lactic acid treatment yielded highly purified hiPSC-CMs

**Figure 2:**
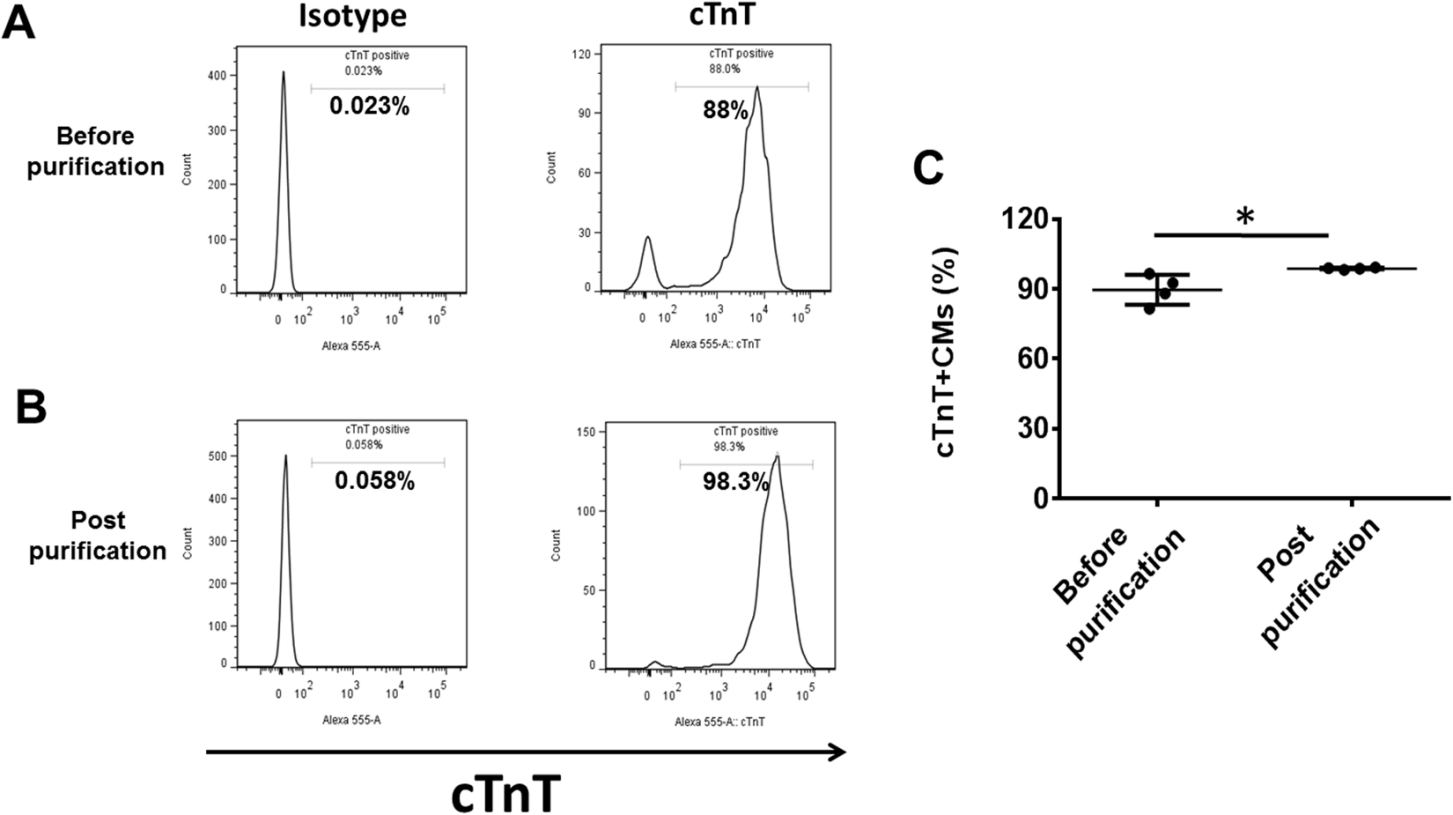
Purity of hiPSC-CMs as determined by flow cytometry. Typical flow cytometry analysis results for cardiac troponin T (cTnT) expression in hiPSC-CMs on day 7 of contraction (before purification) **(A)** and on day 13 of contraction (post purification) after lactic acid treatment **(B)**. **(C)** Mean hiPSC-CMs differentiation and purification efficiencies. (Data are represented as mean ± SD; *p < 0.05).

The CM differentiation efficiency of the protocol was 89.7% ± 6.4% based on cardiac troponin T protein (cTnT) expression by flow cytometry (Fig. 2). A 6-day treatment with lactic acid significantly increase hiPSC-CMs purity to 98.8 ± 0.3% based on cTnT expression (Fig. 2).

### Transfection of hiPSC-CMs with polyplex-pGFP

**Figure 3:**
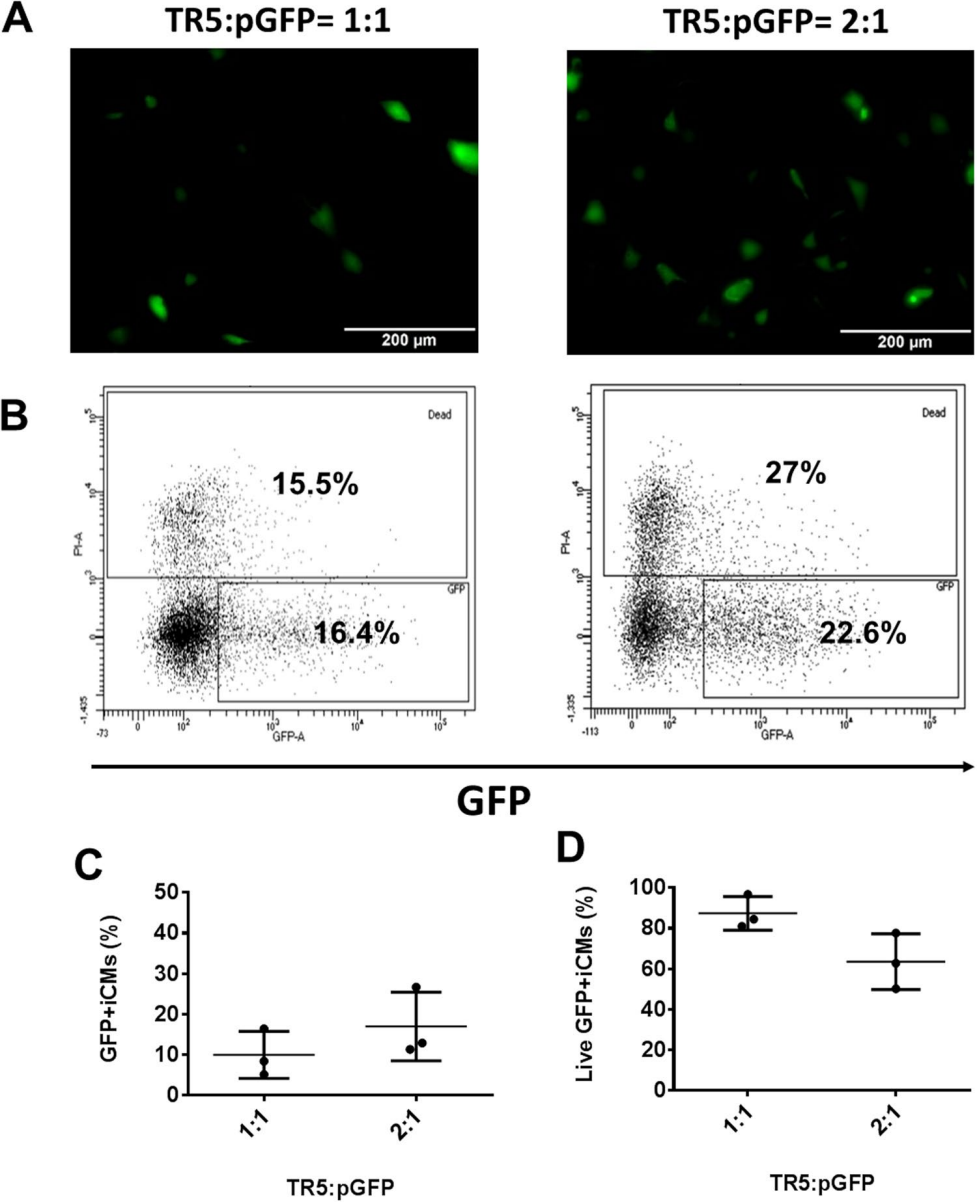
TR5 based pGFP transfection with hiPSC-CMs at transfection ratios of 1:1 and 2:1 on day 16 of contraction. (**A**) Representative images of GFP expression in hiPSC-CMs on day 16 of contraction. **(B)** Representative flow cytometry results to show transfection efficiency and cell death. Mean transfection efficiency **(C)** and cell viability **(D)** of TR5 based hiPSC-CMs transfection with pGFP. (Data are represented as mean ± SD).

First, we determined the TR5 based pGFP transfection with hiPSC-CMs (Fig. 3). The transfection ratio was based on the volume (μl) to quantity of pGFP (μg) according to datasheet of TR5. The transfection efficiency was 9.9 ± 5.8% when 1 μl TR5 was used to encapsulate 1 μg pGFP and the cell viability was 87 ± 8.3%. Though, increasing TR5 to 2 μl improved gene transfection efficiency to 17 ± 8.5%, the cell viability was reduced to 64 ± 13.8%.

**Figure 4:**
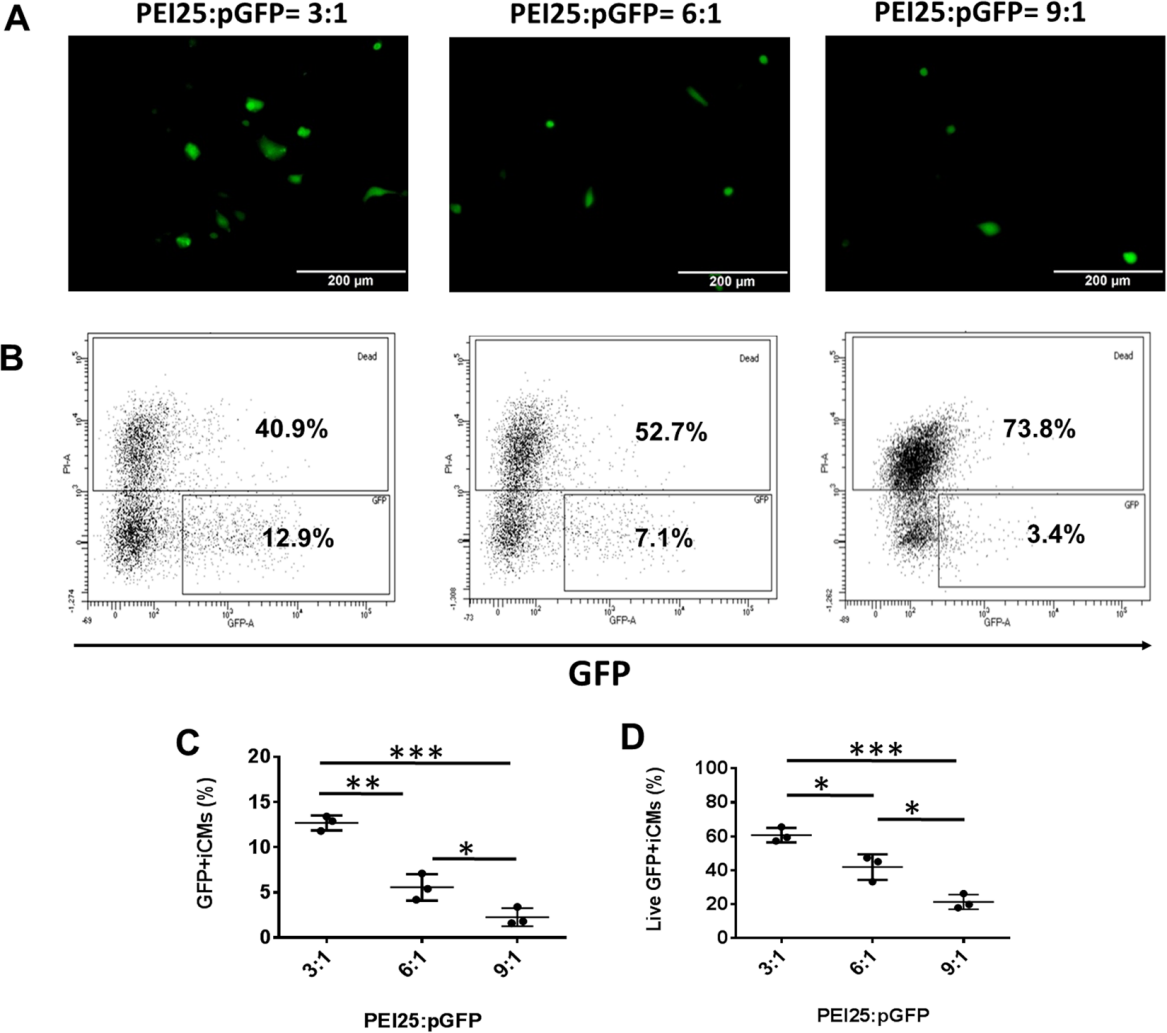
PEI25 based pGFP transfection with hiPSC-CMs at transfection ratios of 3:1, 6:1, and 9:1 on day 16 of contarction. (**A**) Representative images of GFP expression in hiPSC-CMs on day 16 of contraction. **(B)** Representative flow cytometry results to show transfection efficiency and cell death. Mean transfection efficiency **(C)** and cell viability **(D)** of PEI25 based hiPSC-CMs transfection with pGFP. (Data are represented as mean ± SD).(*p < 0.05, **p < 0.01, and ***p < 0.001).

Next, we determined the PEI25 based pGFP transfection with hiPSC-CMs (Fig. 4). The transfection ratio was based on the equivalents of PEI nitrogen per DNA phosphate (N/P)^10,11^. The transfection efficiency was 12.7 ± 1% when N: P ratio was 3:1 and the cell viability was 61 ± 5.8%. However, with further increase of N:P ratio to 6:1 and 9:1, the transfection efficiencies were reduced to 5.6 ± 1.5% and 2.3 ± 1%, respectively. Accordingly, the cell viability was reduced to 42 ± 7.6% and 21 ± 4.3%, respectively.

### Transfection CMs with Lipoplex-pGFP

**Figure 5:**
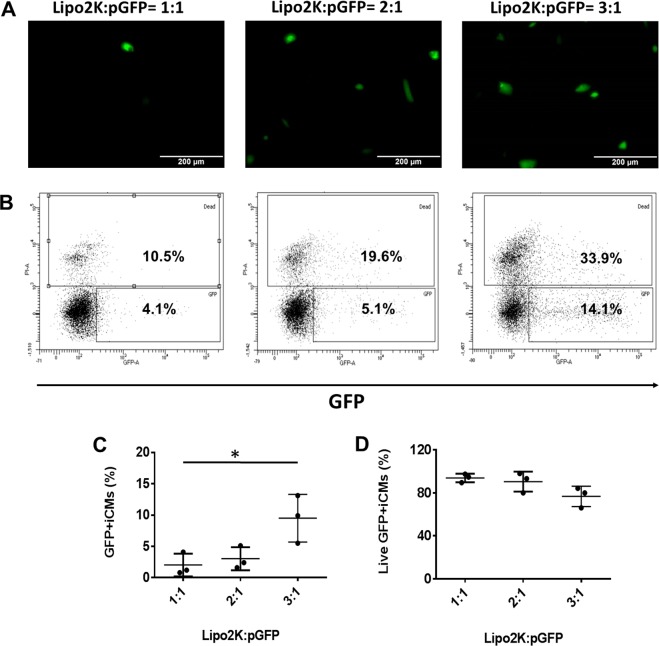
Lipo2K based pGFP transfection with hiPSC-CMs at transfection ratios of 1:1, 2:1, and 3:1 on day 16 of contraction. (**A**) Representative images of GFP expression in hiPSC-CMs on day 16 of contraction. **(B)** Representative flow cytometry results to show transfection efficiency and cell death. Mean transfection efficiency **(C)** and cell viability **(D)** of Lipo2K based hiPSC-CMs transfection with pGFP. (Data are represented as mean ± SD) (*p < 0.05).

First, we determined the Lipo2K based pGFP transfection with hiPSC-CMs (Fig. 5). The transfection ratio was based on the volume (μl) to quantity of pGFP (μg) according to datasheet of Lipo2k. The transfection efficiency was 2 ± 1.8% when 1 μl Lipo2k was used to encapsulate 1 μg pGFP and the cell viability was 94 ± 4%. Further increasing Lipo2k to 2 or 3 μl, the transfection efficiency only increased to 3 ± 1.8% or 9 ± 3.2%, respectively. The cell viability was 90 ± 9.3% or 76 ± 9.6%, respectively.

**Figure 6:**
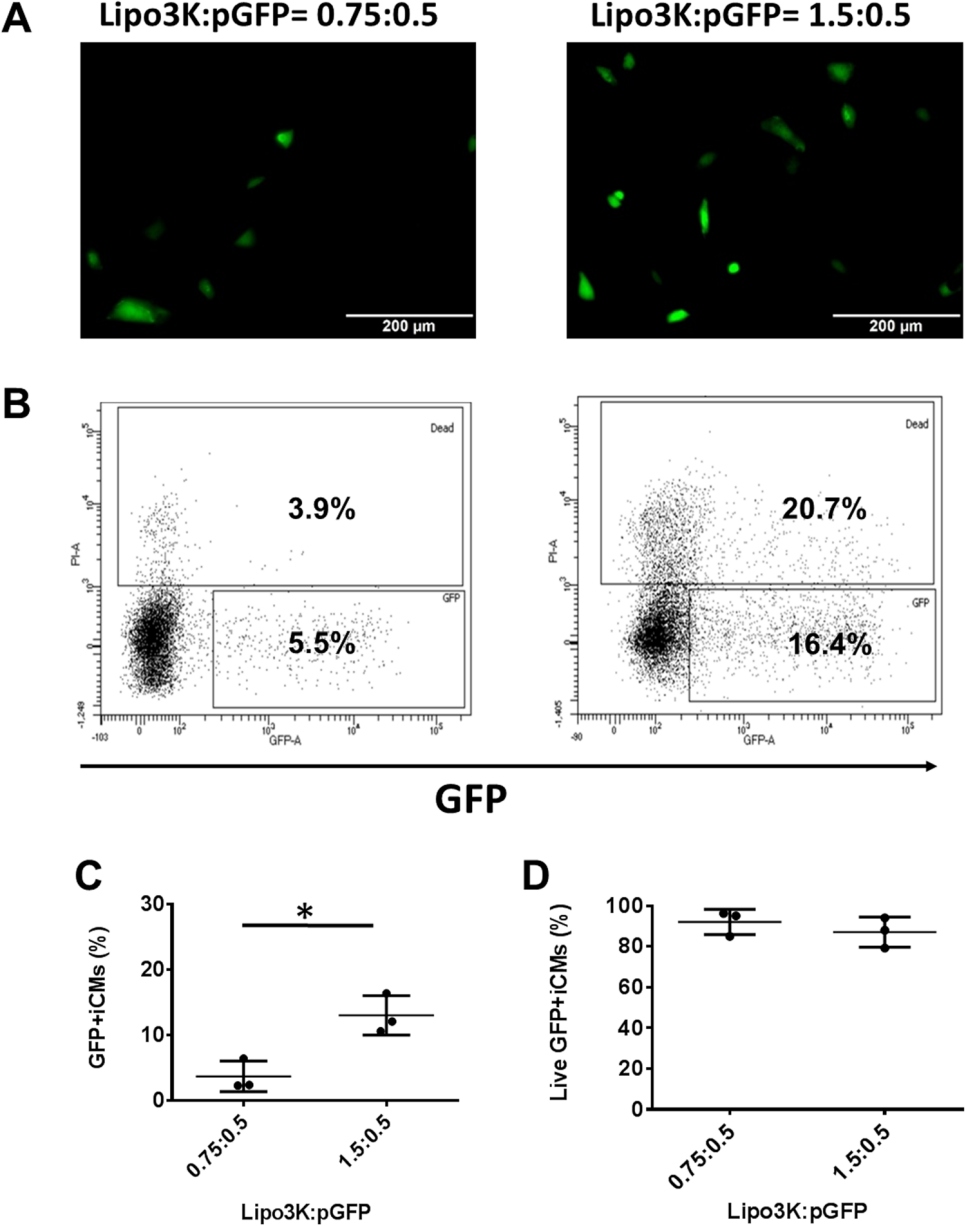
Lipo3K based pGFP transfection with hiPSC-CMs at transfection ratios of 0.75:0.5 and 1.5:0.5 on day 16 of contraction. (**A)** Representative images of GFP expression in hiPSC-CMs on day 16 of contraction. **(B)** Representative flow cytometry results to show transfection efficiency and cell death. Mean transfection efficiency **(C)** and cell viability **(D)** of Lipo3K based hiPSC-CMs transfection with pGFP. (Data are represented as mean ± SD) (*p < 0.05).

Next, we determined the Lipo3K based pGFP transfection with hiPSC-CMs (Fig. 6). The transfection ratio was based on the volume (μl) to quantity of pGFP (μg) according to datasheet of Lipo3k. The transfection efficiencies were 3.7 ± 2.3% when 0.75 μl Lipo3k was used to encapsulate 0.5 μg pGFP and the cell viability was 92 ± 6%. Further increased Lipo3k to 1.5 μl, the transfection efficiency increased to 13 ± 3% and cell viability was 87 ± 7.4%.

**Figure 7:**
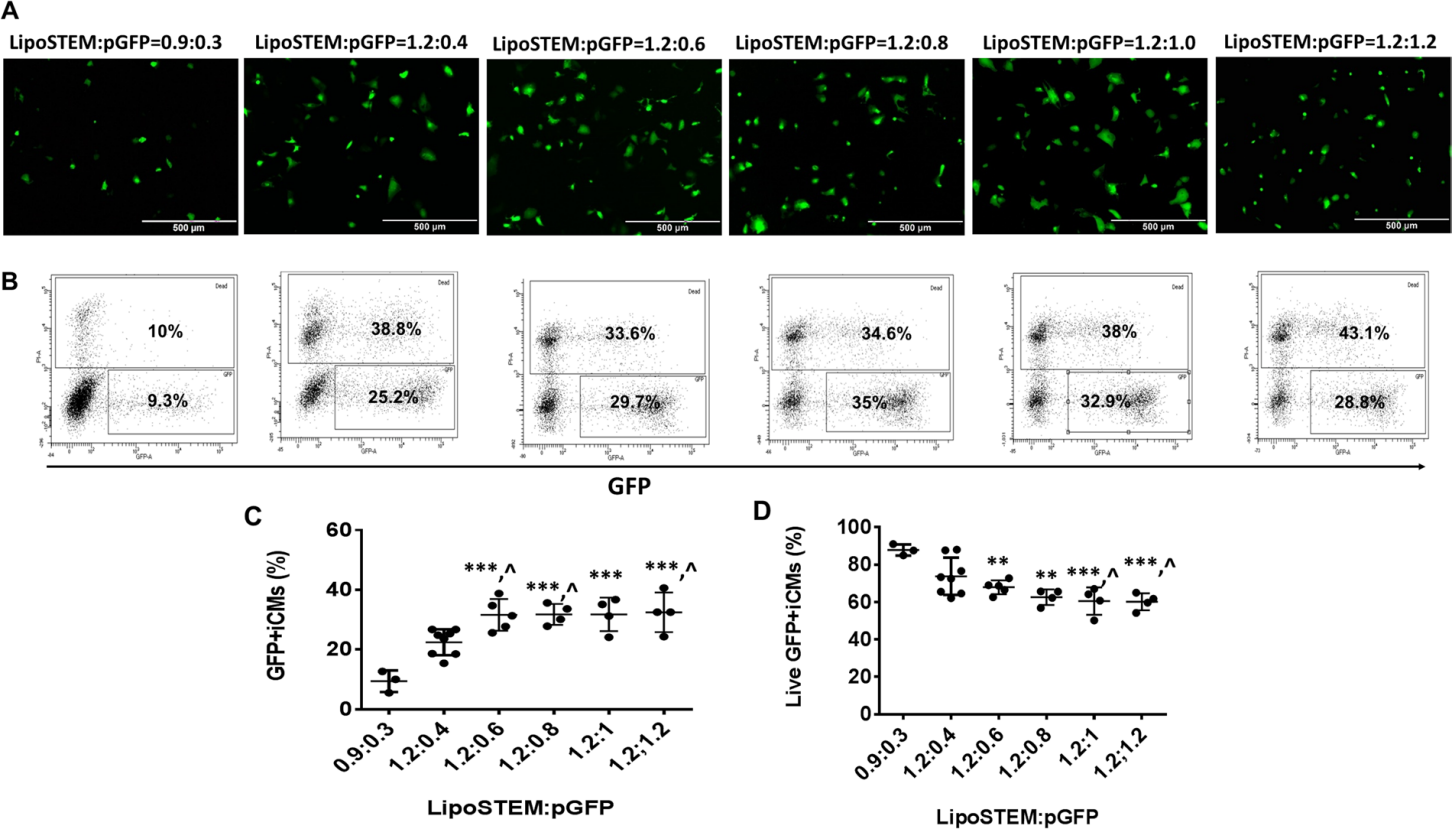
LipoSTEM based pGFP transfection with hiPSC-CMs at transfection ratios of 0.9:0.3, 1.2:0.4, 1.2:0.6, 1.2:0.8, 1.2:1, and 1.2:1.2 on day 16 of contraction. (**A)** Representative images of GFP expression in hiPSC-CMs on day 16 of contraction. **(B)** Representative flow cytometry results to show transfection efficiency and cell death. Mean transfection efficiency **(C)** and cell viability **(D)** of LipoSTEM based hiPSC-CMs transfection with pGFP. (Data are represented as mean ± SD).(** vs 0.9:0.3: p < 0.01, *** vs 0.9:0.3: p < 0.001, and ^ vs 1.2:0.4: p < 0.05).

Thirdly, we determined the LipoSTEM based pGFP transfection with hiPSC-CMs (Fig. 7). The transfection ratio was based on the volume (μl) to quantity of pGFP (μg) according to datasheet of LipoSTEM. We tested the transfection efficiency at ratios of 0.9:0.3 and 1.2:0.4. The transfection efficiency was 9.4 ± 3.6% and 22.5 ± 4.3%, respectively, with cell viability at 87.9 ± 3% or 74 ± 10%, respectively. Next, we fixed LipoSTEM at 1.2 μl and increased pGFP from 0.6 to 1.2 μg, the transfection efficiency only increased to 31.6 ± 5.3%, 31.8 ± 3.5%, 31.8 ± 5.6%, and 32.5 ± 6.7%, respectively. However, the cell viability dropped significantly to 68 ± 3.7%, 62.6 ± 4.1%, 60.6 ± 7.4%, and 60.2 ± 4.5%, respectively.

### Prolonged culture of hiPSC-CMs increased LipoSTEM mediated pGFP gene transfection and cell viability

**Figure 8:**
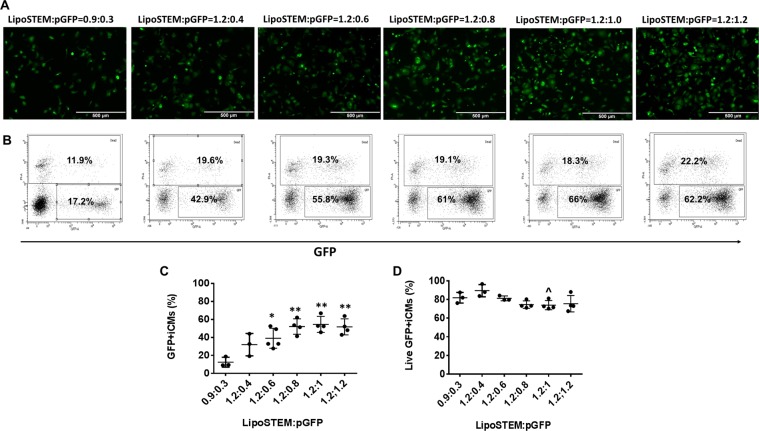
LipoSTEM based pGFP transfection with hiPSC-CMs at transfection ratios of 0.9:0.3, 1.2:0.4, 1.2:0.6, 1.2:0.8, 1.2:1, and 1.2:1.2 on day 25 of contraction. (**A)** Representative images of GFP expression in hiPSC-CMs on day 25 of contraction. **(B)** Representative flow cytometry results to show transfection efficiency and cell death. Mean transfection efficiency **(C)** and cell viability **(D)** of LipoSTEM based hiPSC-CMs transfection with pGFP. (Data are represented as mean ± SD) (** vs 0.9:0.3: p < 0.01, *** vs 0.9:0.3: p < 0.001, and ^ vs 1.2:0.4: p < 0.05).

To determine whether a prolonged culture of hiPSC-CMs can improve LipoSTEM mediated gene transfection and cell viability, hiPSC-CMs were transfected on day 23 of contraction (Fig. 8). It is interesting that prolonged culture of hiPSC-CMs improved LipoSTEM mediated gene transfection efficiency and cell viability: the transfection efficiencies for ratio at 0.9:0.3 and 1.2:0.4 were 12.5 ± 5.5 and 32.1 ± 12.3%, respectively, with cell viability at 81.9 ± 5.7% and 89.5 ± 6.6%, respectively. When pGFP increased from 0.6 to 1.2 μg, the transfection efficiency increased from 40.6 ± 12.1% to 52.1 ± 8.6%, 54.5 ± 8.9%, and 51.7 ± 8.9%, respectively. The cell viability rates were 81.3 ± 2.6%, 74.5 ± 4%, 74 ± 4.9%, and 75.6 ± 8.8%%, respectively.

### Optimized gene transfection protocol efficiently transfected two additional hiPSC lines, P1C1 and DP3, with pGFP

**Figure 9:**
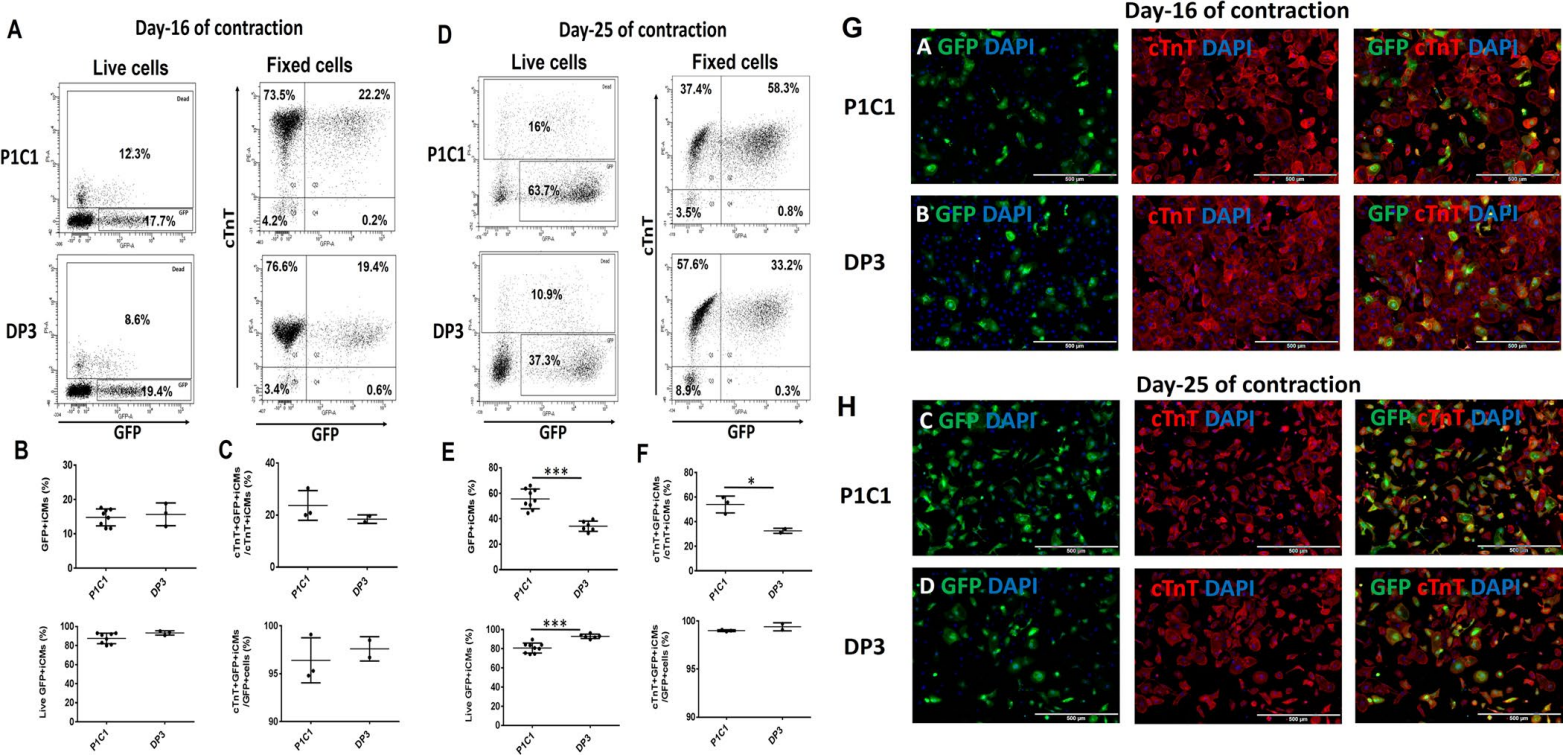
LipoSTEM based pGFP transfection with hiPSC-CMs of P1C1 or DP3 at transfection ratios 1.2:1 on day 16 or 25 of contraction. (**A)** Representative images of GFP expression in live or fixed hiPSC-CMs on day 16 of contraction. **(B)** Mean transfection efficiency and mean cell viability of live hiPSC-CMs on day 16 of contraction. **(C)** Mean transfection efficiency and percentage of GFP + hiPSC-CMs/total GFP + cells on day 16 of contraction after fixation. **(D)** Representative images of GFP expression in live or fixed hiPSC-CMs on day 25 of contraction. **(E)** Mean transfection efficiency and mean cell viability of live hiPSC-CMs on day 25 of contraction. **(F)** Mean transfection efficiency and percentage of GFP + hiPSC-CMs/total GFP + cells on day 25 of contraction after fixation. Representative pictures of GFP expressing hiPSC-CMs stained positive for cTnT on day 16 **(G)** or 25 **(H)** of contraction. (Data are represented as mean ± SD) (*p < 0.05 and ***p < 0.001).

To further determine whether the optimized LipoSTEM medicated pGFP transfection protocol can be applied to other hiPSC lines, P1C1 and DP3 hiPSC lines were tested. On the day 16 of contraction, the mean pGFP gene transfection efficieny was 14.8 ± 2.5% or 15.7 ± 3.3% and cell viability was 87.3 ± 5.5% or 93.3 ± 2.3%, respectively, for live hiPSC-CMs of P1C1 or DP3 (Fig. 9A,B). After fixation, the cTnT + GFP + hiPSC-CMs was 23.7 ± 5.8% or 18.5 ± 1.6%, respectively, for P1C1 or DP3. More than 95% of GFP + cells were cTnT + hiPSC-CMs (Fig. 9A,C).

On the day 25 of contraction, the mean pGFP gene transfection efficieny was 55.6 ± 7.8% or 34.1 ± 4% and cell viability was 80.8 ± 5.2% or 92.9 ± 2.2%, respectively, for live hiPSC-CMs of P1C1 or DP3 (Fig. 9D,E). After fixation, the cTnT + GFP + hiPSC-CMs was 54 ± 6.9% or 32.6 ± 2.1%, respectively, for P1C1 or DP3 and more than 99% of GFP + cells were cTnT + hiPSC-CMs (Fig. 9D,F).

To visualize GFP expressing hiPSC-CMs, pGFP transfected hiPSC-CMs of P1C1 or DP3 were fluorescence immunostained for cTnT expression as shown in Fig. 9G,H. More than 95% GFP expressing cells were stained positive for cTnT.

### Optimized gene transfection protocol efficiently transfected hiPSC-CMs with pAng-1

hiPSC-CMs were transfected with pAng-1 based on the optimized gene transfection protocol using LipoSTEM. The ratio is LipoSTEM:pAng-1 = 1.2 μl:1 μg pAng-1. The human Ang-1 ELISA showed that the mean Ang-1 protein concentration was 29.17 ± 11.8 ng/ml on day 2 post transfection.

## Discussion

Currently, the *ex vivo* delivery of gene into mammalian CMs is mainly successful through viral vectors, including adenovirus, adeno-associated virus, and lentivirus, which yield high gene transduction efficiencies^3,5–9^. Non-viral vector based gene transfection with CMs is very challenging. The current study investigated non-viral vector based pGFP transfection with hiPSC-CMs and showed that LipoSTEM achieved highest GFP gene transfection efficiency in normal hiPSC-CMs (>50%) or diabetic hiPSC-CMs (>30%).

Overall, the highest gene transfection efficiency is achieved by LipoSTEM, followed by TR5, Lipo3K, PEI25, and Lipo2K. It is known that gene transfection efficiency of non-viral vector–mediated gene transfer are influenced by zeta potential, plasmid DNA size, and vector material.

The zeta potentials of TR5 and PEI25 would be <30 mV according to a previous study by our group, and it should be >40 mV for lipoplexes^10,11^. Although a higher zeta potential is preferred for efficient gene transfection, it is not a crucial factor that determines gene transfection efficiency in this study. Since the same pGFP plasmid was used in the study, vector material is the key factor that affects the gene transfection efficiency.

Lipoplexes and polyplexes enter into cells and nuclei through different pathways. Lipoplexes are taken up through clathrin mediated endocytosis^21^. The negatively charged lipid phosphatidylserine destabilize the bilayer membrane organization after interacts with the cationic lipid, which causes competitive dissociation of DNA from the lipoplex and its release into the cytosol^22^. However, polyplexes are taken up through either clathrin- or caveolae-mediated endocytosis. It is caveolae-mediated route that leads to efficient transfection^21^. It was shown that that polyplex DNA released from the endosome is a result of osmotic bursting which was caused by an excessive influx of protons^21^. Thus, lower transfection efficiencies associated with Lipo2K and Lipo3K lipoplexes were observed as compared with TR5 transfection with hiPSC-CMs. A better transfection efficiency of TR5 than that of PEI25 is due to the fact that PEI25 polyplexes can only translocate plasmid DNA into nuclei mainly during the S/G2 phase of CM mitosis, whereas linear PEI (TR5) can translocate plasmid DNA into nuclei independent of CM mitosis or cytokinesis^23,24^. One concern associated with plasmid transfection is that plasmid DNA can integrate into host host genomic DNA, which may cause unknown side-effects due to long-term low expression of transfected gene^25^.

Surprisingly, we found LipoSTEM achieved a better gene transfection efficiency which was increased by 37.6% or 166% as compared with TR5. LipoSTEM has superior transfection efficiency in human embryonic stem cells (ESC), iPSC, and neural stem cells (NSC), and mesenchymal stem cells (MSC) (according to online information provided by Thermo Fisher). However, its effect on hiPSC-CMs is unknown. The current study demonstrates that LipoSTEM has superior transfection efficiency on hiPSC-CMs as compared with Lipo2K, Lipo3K, TR5, and PEI25. It may be a good non-viral vector for gene transfection with cells differentiated from pluripotent stem cells, especially for hiPSC-CMs or skeletal muscle cells which are difficult cells to be transfected with non-viral vectors.

We used suspension hiPSC-CMs for gene transfection as we found that suspension improved liposome or PEI-polymer mediated gene transfection efficiency in a previous study which showed this physical procedure improved transfection efficiency up to 60%^10^. The associated mechanism is unknown. Further study is needed to clarify the mechanism by which trypsin pretreatment enhanced the efficiency of non-viral vector–mediated gene therapy.

Particularly noteworthy is that prolonged cultured of hiPSC-CMs improved transfection and cell viability. The terminally differentiated hiPSC-CMs are unable to undergo cytokinesis, but they undergo mitosis to form bi-nuclei or poly-nuclei cells in cell culture^26^. The capability of mitosis of hiPSC-CMs may help to improved transfection efficiency associated with LipoSTEM. Another factor may arise from hiPSC-CMs. hiPSC-CMs in the early stage of contraction may be more fragile to the toxicity of liposome than in the later stage. A prolonged cell culture may help maturation of hiPSC-CMs *in vitro*^26,27^.

Generally, polymer and liposome based gene tranfection can only achieve a transient gene and protein expressions around 14 days i*n vitro*^10,11^. This time window is sufficient and safe for therapeutic angiogenesis for treatment of ischemic heart disease or ischemic limb disease^11,12^, as localized and prolonged over-expression of angiogenic factor has been shown to cause angioma^28^. In addition to polymer or liposome, PEGylated liposomal nanoparticles which have been shown to have high gene transfection with HEK293 *in vitro*^29^, may have the potent to transfect hiPSC-CMs efficiently.

In conclusion, we determined non-viral vectors, including PEI and liposome, based transfection with hiPSC-CMs using pGFP as a gene model and found that LipoSTEM achieved the highest pGFP gene transfection efficiency with hiPSC-CMs: the highest GFP gene transfection efficiency in normal hiPSC-CMss was >50% or in diabetic hiPSC-CMs was >30%. Further studies should be performed in preclinical large animal models to investigate the therapeutic safety and efficacy of LipoStem mediated angiogenic gene transfection with hiPSC-CMs.

## Data Availability

The data are available upon request.
